# Au⋅⋅⋅H−X (X=N or C) Intramolecular Interactions in Gold (I)‐NHC Carbene Complexes with Potential Anticancer Properties: A Quantum Mechanical Study with Two Basis Sets

**DOI:** 10.1002/open.202400140

**Published:** 2024-06-25

**Authors:** Maria Benavides, Elizabeth Granda

**Affiliations:** ^1^ Department of Natural Sciences University of Houston-Downtown One Main Street Houston Texas 77002

**Keywords:** Gold, Heterometallic Complexes, Density Functional Calculations, Carbene Ligands, Bioinorganic Chemistry

## Abstract

Three cationic Gold(I)‐NHC complexes with potential anticancer properties were studied using DFT with B3LYP functional in combination with two basis sets, LanL2DZ and SDD. Obtained equilibrium geometries and computed IR spectra were found in excellent agreement with previously reported x‐ray structures and experimental IR spectral data. NBO population analysis showed gold(I) has a charge deficiency of 0.26–0.30 e. All three complex cations are polar, with dipole moment values ranging from 6.8 to 7.4 Debye. Regardless of some structural differences in their co‐ligands, all three complex cations have remarkably similar HOMO‐LUMO energy gaps, with values ranging from 5.2 to 5.4 eV, confirming they are chemically stable and that they share an almost identical stability. Long‐range intramolecular interactions Au ⋅⋅⋅H−X (X=N or C) in all three cationic complexes were identified. Both basis sets employed in this study were found equally effective in producing reliable results.

## Graphical abstract

Structural, electronic, and vibrational spectroscopic analyses of three potential anticancer gold(I) NHC complexes with Au⋅⋅⋅⋅H−X (X=N or C) intramolecular interactions using DFT/B3LYP method in combination with two equally effective basis sets that account for relativistic effects that produced equilibrium geometries and computed IR spectra in excellent agreement with x‐ray structures and experimental IR spectral data.

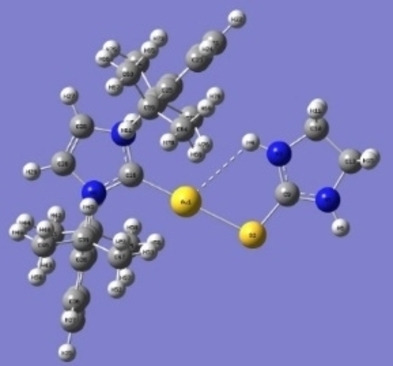

## Introduction

There is considerable interest in transition metal complexes due to their many biological and nonbiological applications, which include antitumor activity.^[1]^ Platinum‐based drugs, like cisplatin and its derivatives, are well known to be effective for their antitumor properties, so they are widely used as chemotherapy agents; but have multiple side effects such as nephrotoxicity, neurotoxicity, ototoxicity, gastrointestinal toxicity, peripheral neuropathy, hematologic toxicity, and can even result in drug resistance.[^2^, ^3^, ^4^, ^5^, ^6^] This has triggered a massive effort into designing and synthesizing alternative metal‐based drugs that can served as effective anticancer agents but that have lower side effects. N‐heterocyclic (NHC) carbene complexes containing Au(I), Ag(I) and Cu(I) are being investigated for their potential role as anticancer agents.^[7]^ However, there is special interest in gold complexes due to their physicochemical properties, which include chemical stability and solubility in physiological environments.^[8]^ Gold compounds generally exhibit high redox potentials due to their significant relativistic effects. Strong σ‐donating ligands, such as NHC carbenes are known to improve the stability of such gold complexes against reduction.^[9]^ When designing metal‐based drugs, apart from the choice of the transition metal, the choice of co‐ligands is a very important consideration because ligands have a crucial role for effectiveness, selectivity, and potency. Nitrogen‐containing heterocyclic rings such imidazole‐like structures, have been chosen for many potent therapeutic agents and common anticancer drugs. This is because of their unique properties, such as high polarity and the ability to hydrogen bond and to coordinate, which allows them to interact with a broad range of biomolecules.^[10]^ Consequently, gold(I)‐NHC carbene complexes that contain imidazole‐like rings are being considered as potential replacement for platinum‐based complexes due to their stability, catalytic properties, controllable steric hindrance, and antitumor potential.^[8]^ Park et al.^[11]^ recently reported a study of a dinuclear gold(I) bis‐NHC imine complex that contains an strongly electron‐donating ligand N‐heterocyclic imine ligand that did not contain inter‐ nor intramolecular aurophilic interactions which was tested for in‐vitro anticancer proliferation against various cells and they found this complex to be highly selective against A549 lung carcinoma. Gulzar et al.^[12]^ recently synthesized and characterized five gold(I)‐NHC carbene complexes that have imidazole‐like rings that were evaluated for in‐vitro anticancer activity against three different cancer cells, using cisplatin as a reference for comparison purposes. The three cancer lines included colon (HTC‐15), lung (A549) and breast (MCF7). Their results indicate that three of the five complexes showed anticancer activity, although with lower effectiveness than cisplatin. Nevertheless, their effectiveness was comparable with previous reports of other gold(I)‐NHC complexes.^[13]^ According to Gulzar et al.^[12]^ the lower effectiveness as anticancer agents could be attributed to steric hindrance involving isopropyl groups in the NHC ligand directly coordinated to gold(I); where gold is also coordinated by a soft thione co‐ligand expected to provide additional stability to the complexes.

To gain greater insight and shed more light into the chemical nature of these complexes and how their structures play a role in their effectives, in this study we employed a quantum mechanical approach to explore the nature of the three complexes that showed some promise as anticancer agents and whose x‐ray crystal structures were reported.^[12]^ The three complexes are: (a) 1,3‐bis(2,6‐diisopropylphenyl)N,N’‐dimethyl‐1,3‐imidazolidine‐2‐thione gold(1) hexafluorophosphate, [Au(IPr)(Me_2_ImS)]PF_6_; (b) (1,3‐bis(2,6‐di‐isopropylphenyl)‐2,3‐dihydro‐1H‐imidazol‐2‐ylidene)‐(1‐ethylimidazolidine‐2‐thione)‐gold(i) hexafluorophosphate, [Au(IPr)(EtImS)]PF_6;_ and (c) (1,3‐bis(2,6‐di‐isopropylphenyl)‐2,3‐dihydro‐1H‐imidazol‐2‐ylidene)‐(imidazolidine‐2‐thione)‐gold(i)hexafluorophosphate, [Au(IPr)(ImS)]PF_6_. In this manuscript, we will use the notation *Au(IPr)* to represent 1,3‐bis(2,6‐diisopropylphenyl)imidazol‐2‐ylidenegold(I), an imidazole ring with two diisopropylphenyl groups attached to it, bonded to Au(I). For the co‐ligands coordinated to gold(I), we will use *ImS* to represent the 1,3‐Imidazolidine‐2‐thione ring; *Me_2_ImS* to represent N,N’‐dimethyl‐1,2‐imidazolidine‐2‐thione ring; and finally, *EtImS* to represent to N‐ethyl‐1,3‐imidazolidine‐2‐thione ring. In our study we also examine the three complexes for potential hydrogen bonding intramolecular interactions involving gold(I). In all tables and figures we use labels for specific atoms based on the labeling scheme used in our calculations.

## Results and Discussion

### Computational Methods

We employed the ab‐initio method Density Functional Theory (DFT),[^14^, ^15^] with the Becke's three parameter^[16]^ with Vosko et al.^[17]^ local correlation part, abbreviated as B3LYP^[18]^ functional, in combination with the two basis sets, LanL2DZ and SDD. These two basis sets were selected because of their effectiveness for molecules involving transition metal atoms.^[19]^ LanL2DZ has been used to study gold(I) complexes,^[20]^ including similar Gold(I) NHC carbenes complexes,^[13]^ while SDD has been used effectively to study gold(I) phosphine complexes.^[9]^ The core basis functions in both basis sets are replaced by effective core potentials (ECP), that retain some of the outer core electrons while incorporating relativistic effects, which in the case of gold, these later ones are considerable.^[21]^ Although both basis sets are effective, we wanted to assess which one of the two yielded results in better agreement with experimental results, and therefore, determine which one is more reliable for future Au(I) NHC carbene complexes. All calculations were performed using the Gaussian 16 suit of codes.[^22^, ^23^]

The x‐ray crystal structures for the three complexes had been deposited in the Cambridge Crystallographic Centre (CCDC 2090806, CCDC 2090807, and CCDC 2080808). We obtained the x‐ray crystal structures, which after the removal of the counterion PF_6_^−^, were used as initial input files in our geometry optimization calculations. This resulted in cationic complexes with a +1 charge. The removal of the counterion reduced the number of atoms and thus the number of electrons, facilitating the processing of these very computationally intensive calculations without compromising the significance of the study, since it is the cationic complex that plays the role as an anticancer agent. Our calculations consisted of optimization calculations that yielded equilibrium geometries, followed by frequency calculations that yielded harmonic vibrational frequencies that generated IR spectra, and these were followed by NBO population analysis calculations to determine the natural population charges.[^24^, ^25^] QTAIM calculations yielded molecular graphs containing atomic charges, bond critical points (BPC), and Laplacian of electron density relief maps and these were carried out using AIMII software, version 19.10.12.^[26]^

### Equilibrium Geometries and Electronic Properties

**Figure 1:**
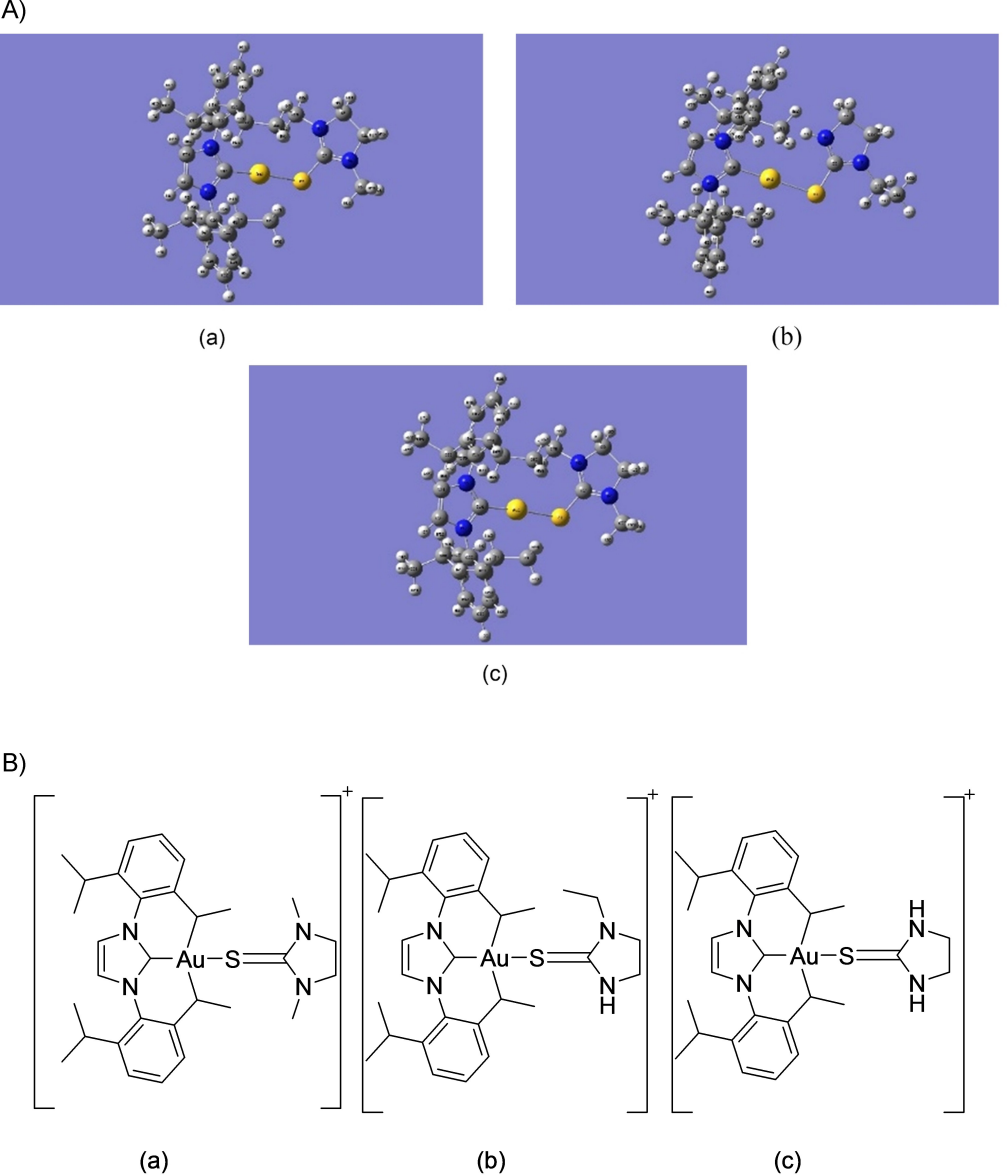
A) Equilibrium geometries of (a) [Au(IPr)(Me_2_ImS)]^+^, (b) [Au(IPr)(EtImS)]^+^and (c) [Au(IPr)(ImS)]^+^. Atom labels correspond to the atom labeling scheme used during calculations. B) The structures of the cationic complexes of (a) [Au(IPr)(Me2ImS)]+, (b) [Au(IPr)(EtImS)]^+^, and (c) [Au(IPr)(ImS)]^+^.

Figure 1A shows the optimized equilibrium geometries for the three gold(I)‐NHC complex ions obtained using the LanL2DZ basis sets, while Figure 1B shows the corresponding structures of the cationic complexes. All three cationic complexes correspond to a singlet electronic state, ^1^A and exhibit similar geometries, apart from some structural differences resulting from the different groups attached to the ImS ring. With respect to the gold(I) coordination sphere, the cationic complexes are not perfectly linear since they are not entirely symmetrical.

Dipole moments for the three cationic complexes are reported in Table 1, with large values ranging from 6.77 to 7.44 Debye. This shows the complexes are polar and therefore expected to be soluble in physiological conditions, which is a desirable property for compounds been considered as candidates for metal‐based drugs.^[8]^ We inspected the Mulliken charges produced in our calculations and found differences between the two basis sets used, whereas the Mulliken charge for S was negative when using the SDD basis sets, it was positive when using the LanL2DZ basis sets. This is not surprising, considering that Mulliken charges can be quite sensitive to the choice of basis sets. Instead, we calculated the NBO charges, which are less dependent on the basis sets and therefore they are more reliable than the Mulliken charges, since they account for bond polarization, and they are based on electron density. Au1 and S2 NBO charges are listed in Table 1. We run the NBO analysis calculations using both basis sets and although there were some minor differences in the NBO charge values between LanL2DZ and SDD basis sets, the trends among NBO charges for all atoms were the same. In all three cationic complexes, Au1 has a positive NBO charge around 0.30 e when LanL2DZ basis sets were used, while SDD basis sets produced slightly less positive values, around 0.26 e. The Au1 positive charge values show that gold is electron deficient, while S2 has a negative charge, indicating that there is a charge transfer from gold to sulfur, when these two coordinate upon the complex formation. This is not surprising considering that gold has a highly polarizable nature with loosely held electrons^[9]^ that facilitate its coordination with S2 in the imidazole‐thione rings, leading to charge deficiency for Au1 and a charge enrichment in the S2 atom. Seliman et al.^[13]^ reported NBO charges for gold of about 0.23 e in other Au(I) carbene complexes containing selenourea, although in their case, the charge transfer occurred between gold and selenium. The NBO charges, also referred as natural charge (NPA), are a significant consideration because charge distributions play a role into how complexes interact with receptor sites in their biological roles. The negative NPA charge in the S2 indicates it has a nucleophilic character, but because its value is not remarkably high, this indicates that it is a soft Lewis base, while Au1 with its low positive NPA charge, corresponds to a soft Lewis acid. The soft thione co‐ligand is expected to contribute to the stability of the complex as bonds form between soft bases and soft acids tend to have a greater covalent character and therefore greater stability; this was one of the considerations of the Gulzar group when designing the gold(I) complexes that are the focus of our study.^[12]^ Laplacian relief maps were obtained for all three cationic complexes and these can be found in the Supporting Information section, Figure S1. The Laplacian relief maps^[27]^ of the cationic complexes are overly complex due to the presence of large number of atoms. These plots provide a topological view of the nucleophilic and electrophilic regions in the complexes. Although the Laplacian plots are complex, it is possible to observe a positive sharp peak on Au1 that indicates there is charge depletion, confirming its nucleophilic nature, although surrounded by a negative peak corresponding due to the lone pairs of the filled d orbitals. S2 has a negative peak that confirms its electrophilic character.

**Table 1:**
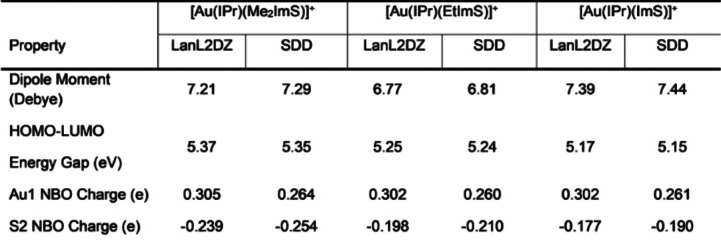
Dipole moments, HOMO‐LUMO energy gaps, and select NBO charges for [Au(IPr)(Me_2_ImS)]^+^, [Au(IPr)(EtImS)]^+^, and [Au(IPr)(ImS)]^+^.

Both the ligand and thione co‐ligands correspond to diaminocarbenes, characterized by a carbon in the carbene center with electron deficiency that is reduced by donation from the two lone pairs on the nitrogen atoms directly bonded to it, whereas its lone pair is stabilized by inductive effects from the two electronegative N atoms that function as π‐donor substituents.^[28]^ The NBO analysis reveals that the C atom in the carbene center of the *Au(IPr)* ^*+*^ system for the three cationic complexes (C atom bonded to Au1), has a NPA charge of about 0.16 e when using LanL2DZ basis sets or 0.18 e when using SDD basis sets. This NPA charge is much smaller as compared to the NPA charge for the C atom in the carbene center (C atom bonded to S2) in the corresponding thione co‐ligands *Me_2_ImS*, *EtImS*, and *ImS* where it has a NPA charge of about 0.37 e when using LanL2DZ basis sets or about 0.40 e when using SDD basis sets. Marchione et al.^[28]^ conducted computational studies on various gold(I)‐NHC complexes and found that π‐back donation is significant in majority of these compounds. NBO second order perturbation analysis reveals dπ–pπ back donation from a lone pair in Au1 corresponding to a filled 5d orbital to an antibonding between N and C located in the NHC ligand directly coordinated to Au1 which corresponds to the (Au(IPr)^+^ system, specifically N5−C14 for [Au(IPr)(Me_2_ImS)]^+^; N6−C15 for [Au(IPr)(EtImS)]^+^; and N7−C16 for [Au(IPr)(ImS)]^+^. This dative bond contributes makes a significant contribution to the stability of these complexes by adding between 11.3 to 11.4 kcal/mol of stabilization to these complexes. This value is comparable to the value reported by Comas‐Vives and Harvey^[30]^ for π dative bonding for an Au(NHC)_2_^+^ complex. This dative bond can help explain why the positive NPA charges in the carbon atom of the carbene center located in the in the NHC ligand in the Au(IPr)^+^ system is smaller as compared to the larger positive NPA charge in the C atom in carbene center of the thione co‐ligands, *ImS*, *Me_2_ImS*, and *EtImS*. The nitrogen atoms in both the ligand directly coordinated to Au1 and the thione co‐ligands have NPA charges around −0.45 e when they correspond to tertiary amines, or around −0.63 e when they correspond to secondary amines, regardless of the choice of basis sets, which confirmed their electronegative nature and their nucleophilic character.

The electron configuration for gold consists of mostly closed shells, [core] 6 s^1^ 5d^10^, which helps to explain why gold(I) can have the lowest coordination number equal to 2. The natural electron configuration of Au1 using LanL2DZ is: [core] 6 s^0.89^ 5d^9.71^ 6p^0.08^ 6d^0.01^, [core] 6 s^0.91^ 5d^9.71^ 6p^0.08^ 6d^0.01^, and [core] 6 s^0.91^ 5d^9.71^ 6p^0.08^ 6d^0.01^ for [Au(IPr)(Me_2_ImS)]^+^, [Au(IPr)(EtImS)]^+^, and [Au(IPr)(ImS)]^+^, respectively. While the natural electron configuration for Au1 when using SDD basis sets is nearly identical, except that it includes a very small contribution from the 7p orbital and it has a little more 6 s character: [core] 6 s^0.92^ 5d^9.70^ 6p^0.08^ 6d^0.01^ 7p^0.01^, [core] 6 s^0.94^ 5d^9.70^ 6p^0.08^ 6d^0.01^ 7p^0.01^, and [core] 6 s^0.94^ 5d^9.70^ 6p^0.07^ 6d^0.01^ 7p^0.01^ for these same complex cations, [Au(IPr)(Me_2_ImS)]^+^, [Au(IPr)(EtImS)]^+^, and [Au(IPr)(ImS)]^+^, respectively.

The Gulzar group^[12]^ used cyclic voltammetry (CV) and square wave voltammetry (SWC) to study the interaction of the gold(I) complexes with L‐tyrosine, and amino acid they used as model to explore the interactions between the complexes and biomolecules. Their results indicate that both [Au(IPr)(EtImS)]^+^, and [Au(IPr)(ImS)]^+^ had a high level of interaction with L‐tyrosine while [Au(IPr)(Me_2_ImS)]^+^ did not appear to interact with the amino acid. The interaction of [Au(IPr)(EtImS)]^+^, and [Au(IPr)(ImS)]^+^ with L‐tyrosine was attributed to the presence of secondary amino groups in the thione co‐ligands which resulted in electrostatic interactions between the complexes and L‐tyrosine. [Au(IPr)(ImS)]^+^ showed the strongest interaction with L‐tyrosine, which is not surprising, considering that it has two secondary groups, while [Au(IPr)(EtImS)]^+^ has just one. [Au(IPr)(Me_2_ImS)]^+^ does not have secondary amino groups, but it does have tertiary amino groups, which appeared to weakly interact with L‐tyrosine. Their results suggest that the secondary amino groups play a role in the interaction between the complexes and L‐tyrosine. Our equilibrium geometries indicate that the secondary amino groups in [Au(IPr)(EtImS)]^+^, and [Au(IPr)(ImS)]^+^, may be experiencing steric hindrance from their proximity to the isopropyl groups in the Au(IPr)^+^ system which could account for the lower performance of these complexes as anticancer agents in comparison to cisplatin or other gold(I) complexes.

**Figure 2:**
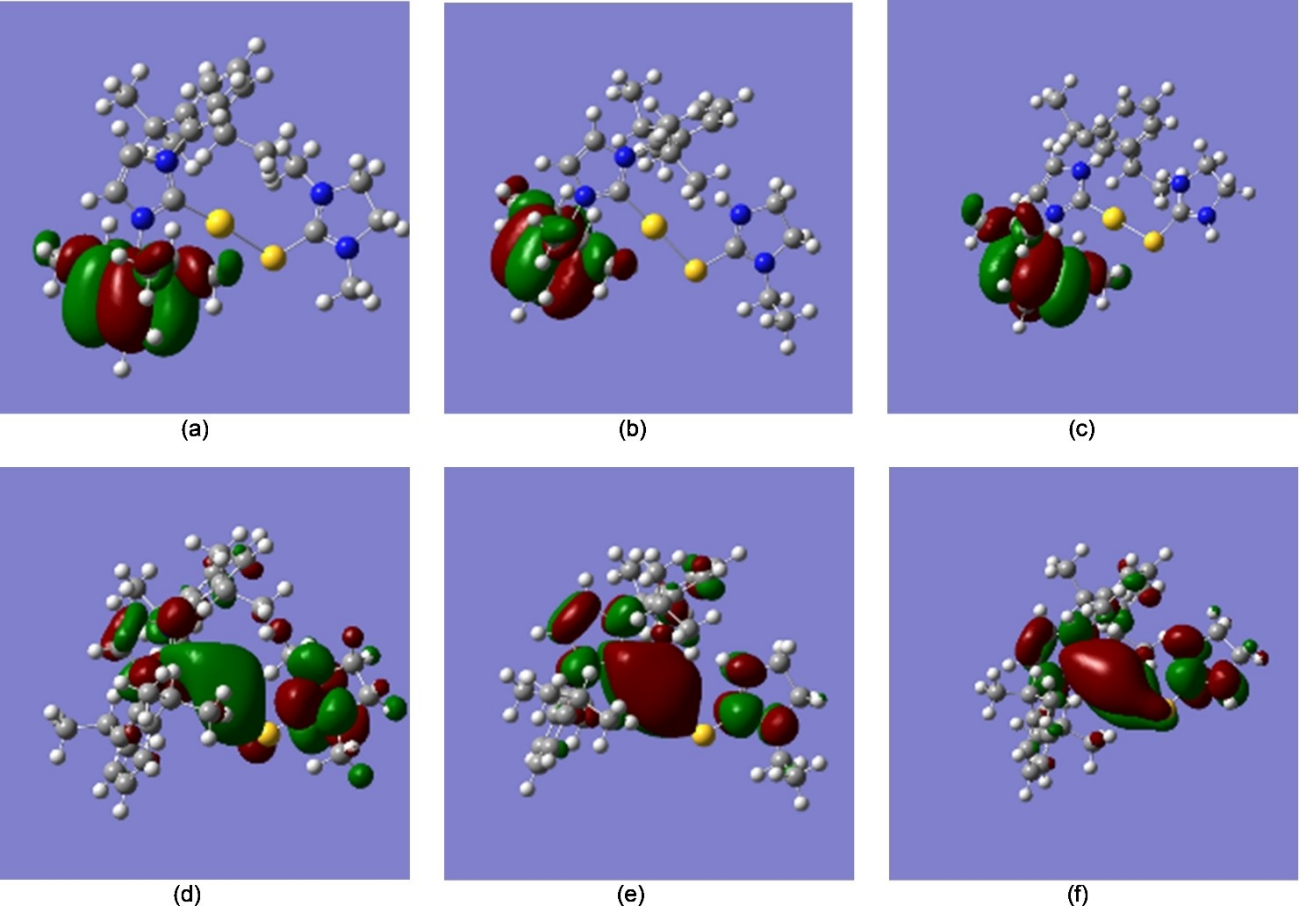
In the top, HOMO orbitals corresponding to (a) [Au(IPr)(Me_2_ImS)]^+^ (b) [Au(IPr)(EtImS)]^+^, and (c) [Au(IPr)(ImS)]^+^ In the bottom, LUMO orbitals corresponding to (d) [Au(IPr)(Me_2_ImS)]^+^, (e) [Au(IPr)(EtImS)]^+^, and (f) [Au(IPr)(ImS)]^+^.

The three cationic complexes have almost identical highest occupied molecular orbital (HOMO) – lowest unoccupied molecular orbital (LUMO) energy gaps, ranging from 5.15 to 5.37 eV, shown in Table 2. The choice of basis sets between LanL2dz and SDD does not seem to have a major effect on the value of this energy gap. These values indicate the complexes are chemically stable, another desirable property for complexes intended to be used as metal‐based drugs where chemical stability in physiological environments as well as shelf life are particularly important considerations. HOMO and LUMO orbitals are shown in Figure 2, and it is interesting to notice that the composition of the HOMO orbitals is remarkably similar across all three cationic complexes, which is also the case among the LUMO orbitals. In all three complex cations, the HOMO orbital is primarily localized in one of the diisopropyl benzyl rings attached to the imidazole ring in the Au(IPr)^+^ system, while the LUMO orbitals are more widely distributed on the imidazole‐2‐thione rings, Au1, including the imidazole ring in the Au(IPr)^+^ system. The similarities among the HOMO orbitals and among the LUMO orbitals helps explain the remarkable similarity among the values of the HOMO‐LUMO energy gaps in all three complexes.

**Table 2:**
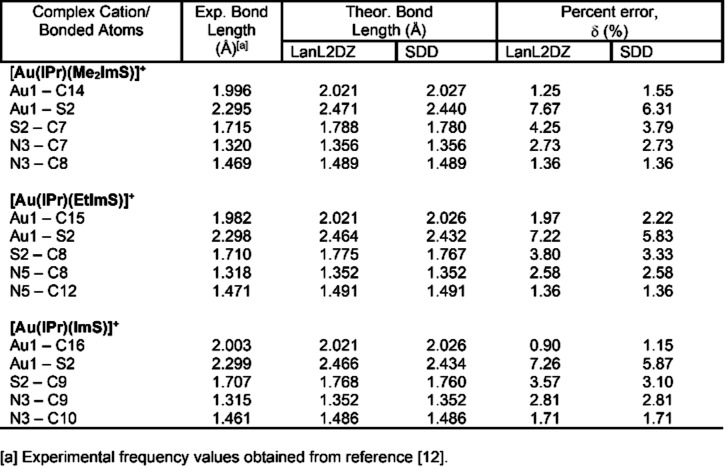
Comparison of select experimental and computed bond lengths of [Au(IPr)(Me_2_ImS)]^+^, [Au(IPr)(EtImS)]^+^, and [Au(IPr)(ImS)]^+^ and error percentages (δ).

We compared our optimized geometries to the x‐ray crystal structures.^[12]^ Table 2 includes a comparison of select experimental and computed bond lengths, while Table 3 includes a comparison of select experimental and computed bond angles, calculated using LanL2DZ and SDD basis sets and with the inclusion of percent errors. When using LanL2DZ basis sets, the percent error for the bond lengths ranges from 0.9 to 7.67 %, while the percent error for bond angles ranges from 0 to 8.3 % which are within the same range that Binbay^[31]^ reported using DFT in combination with LanL2DZ basis sets for a half‐sandwiched Ruthenium complex, which is quite significant, if one considers that Ru does not have the substantial relativistic effects that gold has and we still obtained about the same percent errors. We obtained similar percent errors while using SDD basis sets, which shows these basis sets account for gold's relativistic effects as effectively as LanL2DZ basis sets. Seliman et al.^[13]^ obtained percent errors within the same range in their DFT calculations for several gold(I) carbene complexes when they used LanL2DZ basis sets. Since in our calculations, the complex cations were not subjected to the external constraints of the solvated crystal structures, this allowed the complex cations to relax a bit, allowing them to adopt a more linear conformation with respect to the coordination sphere of Au1 in comparison to the x‐ray crystal structures, which also resulted in a minor elongation of the bond lengths. The elongation of the bond lengths during quantum mechanical calculations is a common occurrence because the calculations are run as in in the gas phase.

**Table 3:**
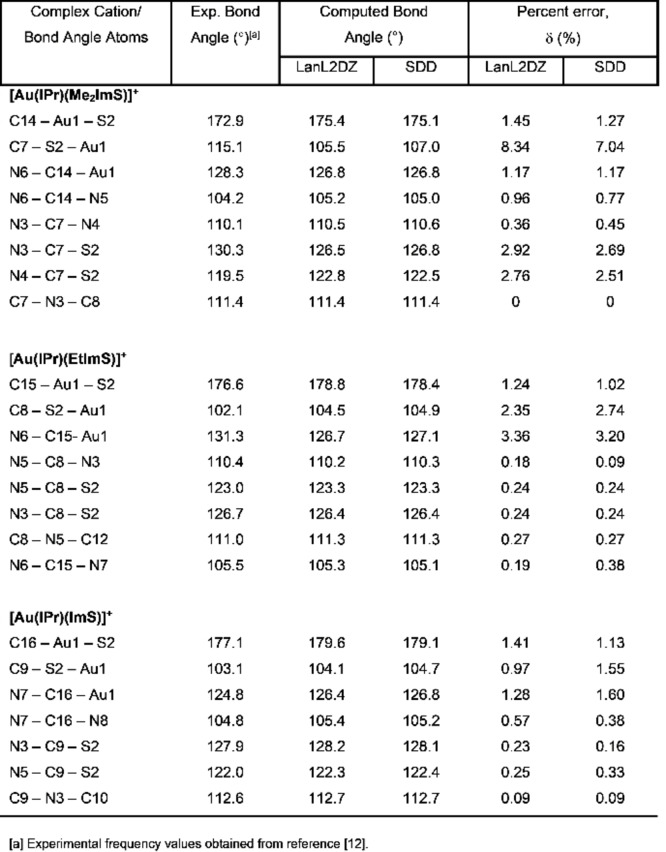
Comparison of select experimental and computed bond angles of [Au(IPr)(Me_2_ImS)]^+^, [Au(IPr)(EtImS)]^+^, and [Au(IPr)(ImS)]^+^ and error percentages (δ).

### Intramolecular Au⋅⋅⋅H−X (X=N or C) Interactions

Although there is great interest in both intra – and intermolecular aurophilic interactions in gold complexes, until few years ago, intramolecular Au⋅⋅⋅H−C and Au⋅⋅⋅H−N interactions were not well recognized nor well understood because it would require to consider the possibility of the existence of hydrogen bonding involving gold, a transition metal.[^32^, ^33^, ^34^] This led to debate and speculation about the nature of these interactions until recent reports provided experimental and computational evidence of their existence.[^35^, ^36^]

In a recent study, Straka et al.^[35]^ reported experimental and computational evidence that indicated the presence of a moderate strength stabilizing Au⋅⋅⋅H^+^−N^−^ intramolecular interaction through hydrogen bonding to gold in a gold(I) complex cation [Cl−Au−L]^+^, where L corresponded to a protonated N‐heterocyclic carbene. They reported the Au⋅⋅⋅H−N interaction to have a distance of 2.14–2.17 Å and they also reported weak Au⋅⋅⋅H−C interactions involving a tert‐butyl group, with a distance of 2.77–2.84 Å. Rigoulet et al.^[36]^ reported the presence of hydrogen bonding Au⋅⋅⋅H−N in cationic gold(I) complexes with ditopic phosphine‐ammonium ligands which they inferred using NMR and IR spectroscopy as well as a computational approach; their reported Au⋅⋅⋅H−N distance was relatively short, corresponding to 2.58 Å, with a wide bond angle of 158°. Aurophilic interactions, Au⋅⋅⋅H−C were also observed by Darmandeh et al.^[37]^ where they report remarkably short distances for these interactions, in the range of 2.36–2.39 Å for gold(I) complexes containing ylide‐phosphine moieties.

**Figure 3:**
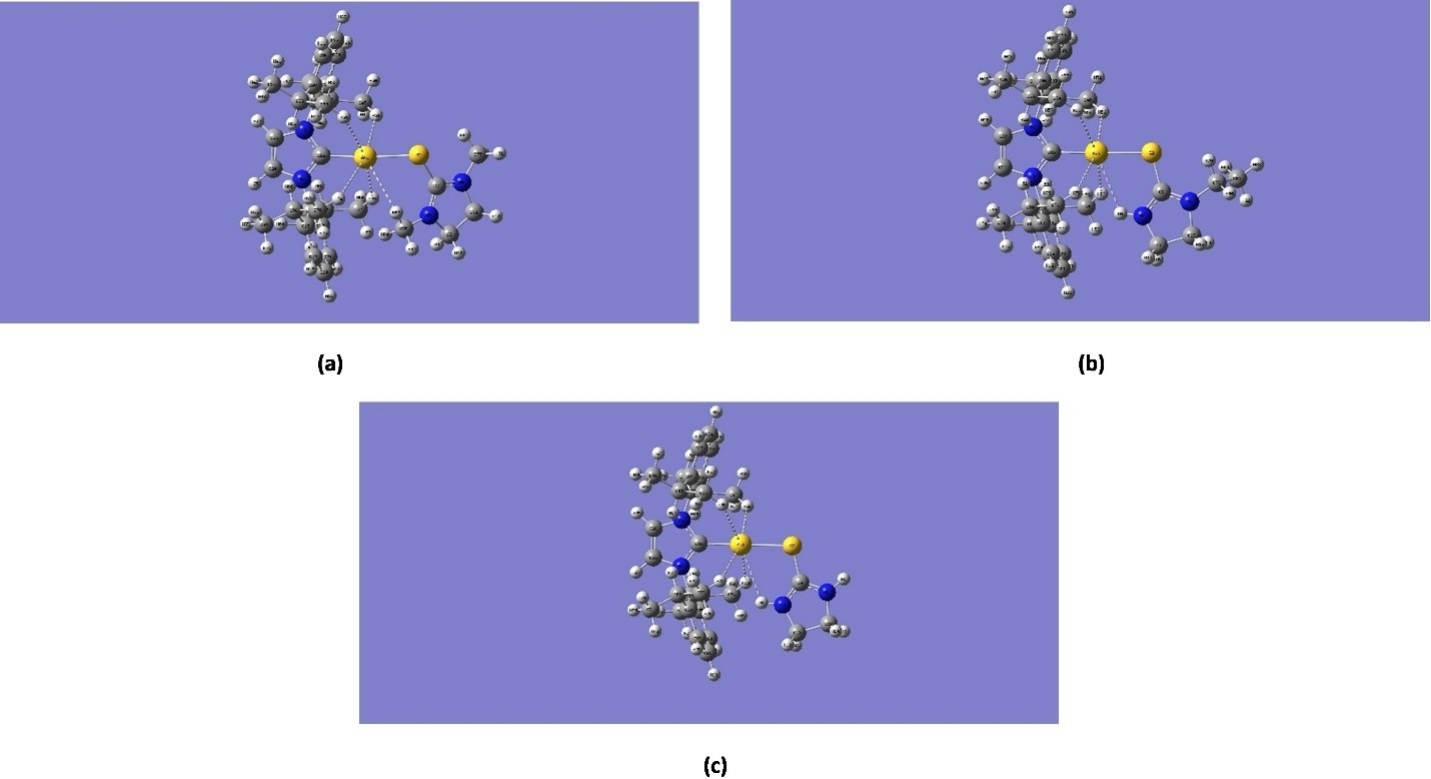
Au⋅⋅⋅H−X (X=N or C) intramolecular interactions are shown with white dashed lines for (a) [Au(IPr)Me_2_ImS]^+^, (b) [Au(IPr)EtImS]^+^, and (c) [Au(IPr)ImS]^+^.

Gulzar et al^[12]^ reported there were no aurophilic interactions in the three cationic complexes that are the focus of our study, although they did not specify if they were referring to intermolecular or intramolecular interactions. Upon close examination of the equilibrium geometries, there appears to be a long‐range intramolecular Au⋅⋅⋅H−N interaction in both [Au(IPr)(EtImS)]^+^ and [Au(IPr)(ImS)]^+^ involving Au1 and H4 from the secondary amino group in the imidazole‐2‐thione ring, with a distance of 2.86 Å and 2.91 Å, respectively; in the corresponding x‐ray crystal structures, the distances are 2.70 Å and 2.86 Å, respectively. In the case of [Au(IPr)(Me_2_ImS)]^+^, there is a longer‐range intramolecular Au⋅⋅⋅H−C interaction between Au1 and H83 from one of the two methyl groups attached to the imidazole‐2‐thione ring, with a distance of 3.16 Å, while in the x‐ray crystal structure, the distance is 3.13 Å. The interactions Au⋅⋅⋅H−X (X=N or C) are represented by white dashed lines in Figure 3.

**Figure 4:**
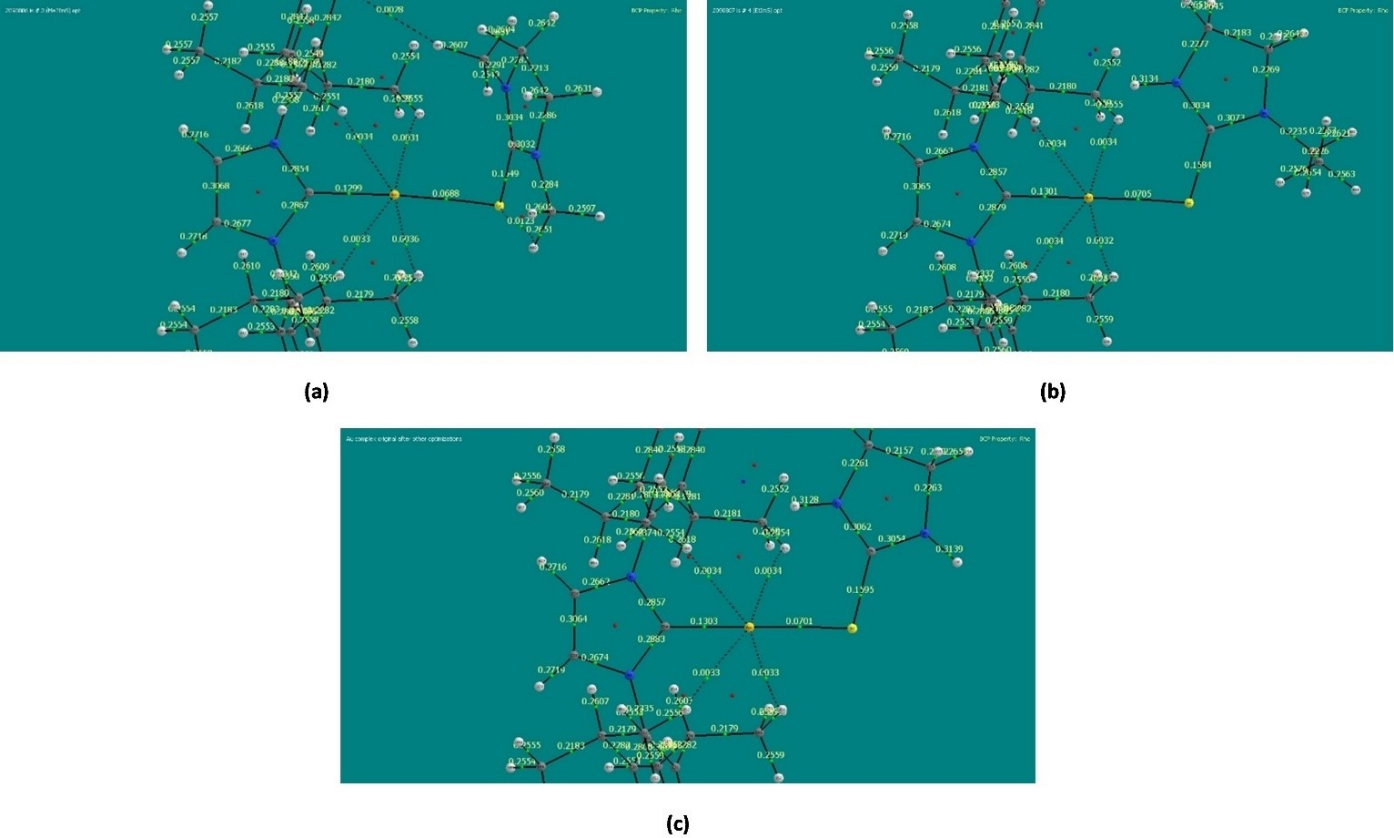
Molecular graphs showing bond critical points (BCP) of (a) [Au(IPr)Me_2_ImS]^+^, (b) [Au(IPr)EtImS]^+^, and (c) [Au(IPr)ImS]^+^.

In addition to the hydrogen bond interactions involving Au1 already discussed, we detected even longer intramolecular interactions between Au1 and four hydrogens, one from each isopropyl group that result from the spatial orientation adopted by the isopropyl groups attached to the benzyl rings bonded to the central imidazole ring of the Au(IPr)^+^ system that maximizes the interactions between Au1 and these four hydrogens. The lengths of these interactions range from 3.34 Å to 3.44 Å in all three cationic complexes; admittedly, the distance between Au1 and these four hydrogen atoms is exceedingly long, although they seem to be akin to those reported by Straka et al.^[35]^ involving a tert‐butyl group and gold(I). Figure 4 shows molecular graphs for the cationic complexes obtained using AIMII software.^[26]^ These graphs include bond critical points (BPC)^[27]^ represented as small green spheres with electron density (rho) values shown in light yellow font color. It is interesting to notice that these molecular graphs indicate they are weak interactions between the same H atoms of the isopropyl groups and Au1 already discussed in terms of Au1⋅⋅⋅H−C interactions, as indicated by black dashed lines, which include bonding critical points corresponding to small electron density values, ranging from 0.0031 to 0.0036 e bohr^−3^ which provide further evidence of the existence of such weak intramolecular interactions. In the Supporting Information section, we have included larger images of the molecular graphs of the cationic complexes that include both atomic charges and bond critical points in Figure S2. On the other hand, these interactions could also be the result of other factors related to the crystallization of these complexes, such as crystal packing. Both intra‐ and intermolecular aurophilic interactions result from gold unique properties, that include being the most electronegative among all metals, having a high electron affinity, and high electrochemical potential, that together with its relativistic contraction, results in gold having a very small atomic and ionic radius and therefore the lowest possible coordination number of 2.^[34]^ Furthermore, noncovalent forces are known to influence the shape of molecules and crystal structures, and aurophilic interactions are not an exception, as recently reported by Pawledzio et al.^[38]^

### Vibrational Spectroscopic Analysis

The vibrational features of the three cationic complexes were investigated by comparing experimental and computed IR spectral data as well as animating the vibrational modes. Computed IR spectra were obtained from frequency calculations that yielded harmonic vibrational frequencies and they are provided in the Supporting Information section, Figure S3. It is known that DFT/B3LYP can provide very reliable computed IR spectra, but because the vibrational frequencies obtained using a quantum mechanical approach are generally higher due to the variational principle, these need to be scaled by factors that depend on the quantum mechanical method used and the choice of basis sets. Although scaling factors have been published for a variety of basis sets used in DFT calculations, empirical scaling is a better approach because it results from the direct comparison of the calculated vibrational frequency values to the experimental ones.^[39]^ Table 4 presents a comparison between select experimental and computed IR spectral data obtained using LanL2DZ and SDD basis sets.

**Table 4:**
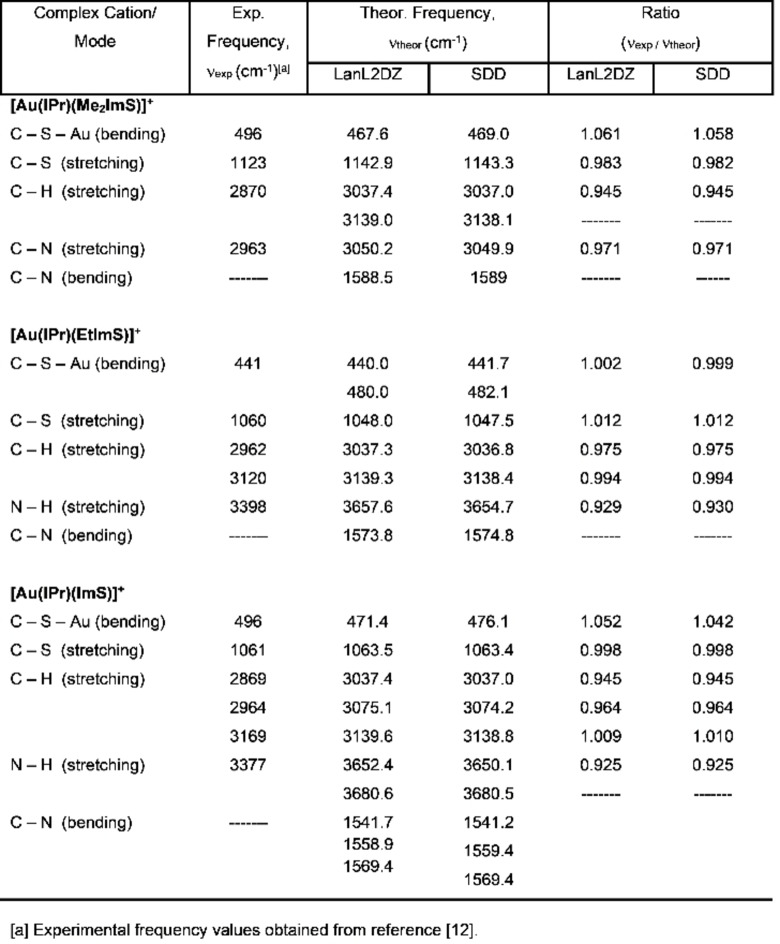
Comparison between select experimental and computed IR spectral data for [Au(IPr)(Me_2_ImS)]^+^, [Au(IPr)(EtImS)]^+^, and [Au(IPr)(ImS)]^+^.

Gulzar et al.^[12]^ reported for all three complexes a ν(C−S−Au) bending mode at low frequency values, either at 441 cm^−1^ or 496 cm^−1^. We identified this low intensity bending mode in the computed IR spectra, and for the case of [Au(IPr)(EtImS)]^+^, it was nearly identical to the experimental value. In the case of the two other cationic complexes, our computed frequency values for this bending mode were slightly lower than the experimental values. In the case of [Au(IPr)(EtImS)]^+^ we also identified an additional, although a slightly more intense ν(C−S−Au) bending mode at 480 cm^−1^ not reported by the Gulzar's group.

Since [Au(IPr)(Me_2_ImS)]^+^ only has tertiary amino groups, it does not have ν(N−H) stretching modes. On the other hand, [Au(IPr)(EtImS)]^+^ has one secondary amino group resulting in one ν(N−H) stretching mode while [Au(IPr)(ImS)]^+^ has two secondary amino groups, resulting in two calculated ν(N−H) stretching modes reported in Table 4. The Gulzar group reports just one single value for the stretching mode for both [Au(IPr)(EtImS)]^+^ and [Au(IPr)(ImS)]^+^, at 3398 cm^−1^ and 3377 cm^−1,^ respectively.

We are also reporting an intense ν(C−N) scissoring bending motion around 1589 cm^−1^ for [Au(IPr)(Me_2_ImS)]^+^ and around 1574 cm^−1^ for [Au(IPr)(EtImS)]^+^ in the Me_2_ImS and EtImS rings, respectively. In the case of [Au(IPr)(ImS)]^+^, there are three bending modes ν( C−N) of medium intensity in the ImS ring, with the most intense been the one at 1569.4 cm^−1^. These bending modes are not reported by Gulzar et al.^[12]^ but are included in Table 4 since we aim to provide a more comprehensible vibrational spectroscopic analysis.

In general, the vibrational modes are concerted with various motions (bending and/or stretching) simultaneously occurring in the complexes, so it is difficult to clearly classified the vibrational modes into specific motion categories. In addition to the modes already discussed, for all three cationic complexes, we observed:

(a) wagging bending motions in the Au(IPr)^+^ group, in the imidazole ring at 791 cm^−1^ and the attached benzyl rings in the 798–855 cm^−1^ region; (b) rocking bending motion of the benzyl rings and the isopropyl groups in the Au(IPr)^+^ group around 957 cm^−1^; (c) scissoring bending motion of the secondary amino groups of the imidazole‐2‐ thione rings at 1222.5 cm^−1^ (ImS) and 1284 cm^−1^(EtImS), not observed in the Me_2_ImS since it has no secondary amine groups; (d) rocking bending motion of the benzyl groups attached to the imidazole of the Au(IPr)^+^ group around 1238 cm^−1^; (e) rocking bending motions of the imidazole‐2‐thione rings, in the regions 1339–1341 cm^−1^ and 1241–1445 cm^−1^; (f) scissoring bending motion of the imidazole‐2‐thione rings in the range 1341–1355 cm^−1^; (g) rocking bending motion of the imidazole ring in Au(IPr)^+^ around 1437 cm^−1^; (h) scissoring bending motion of the imidazole thione rings that resulted in an intense peak in the region 1574–1588.5 cm^−1^; and (i) multiple stretching modes of the isopropyl groups in the 3032–3042 cm^−1^ region, with an intense asymmetric stretching mode at 3139 cm^−1^.

Overall and regardless of the choice of basis, LanL2DZ2 or SDD basis sets, the agreement between the experimental IR vibrational modes and those calculated in this study is excellent. The average ratio of the experimental frequencies to the theoretical frequencies for all three cationic complexes at both basis sets is close to unity, 0.98. This shows that in terms of producing computed IR spectral data, the two basis sets are equally reliable and therefore, either one is a good choice to study gold(I)‐NHC carbene complexes.

## Conclusions

Our study indicates that despite some structural differences in the imidazole‐thione co‐ligands, all three gold(I) complex ions, namely [Au(IPr)(Me_2_ImS)]^+^, [Au(IPr)(EtImS)]^+^, and [Au(IPr)(ImS)]^+^, share similar chemical properties. They possess similar solubility due to their having comparable dipole moment values; similar conformations, as one can observe from a comparison of their bond lengths and bond angles; and very similar chemical stability, as suggested by their nearly identical HOMO‐LUMO energy gap values. Two of the cationic complexes exhibit long‐range Au⋅⋅⋅H−N intramolecular interactions while the third one has an even longer‐range Au⋅⋅⋅H−C intramolecular interaction. And finally, but importantly, both basis sets used in this study are adequate for studying gold(I)‐NHC carbene complexes as they both yield equally reliable results, as demonstrated by the excellent agreement between the experimental and computed IR spectral data, and between the x‐ray crystal structures and our computed equilibrium geometries. The Gulzar group has recently synthesized additional Au(I)‐NHC complexes that were evaluated for in vitro cytotoxicity, we plan to conduct theoretical studies on these new complexes as well.^[40]^

## Conflict of Interests

The authors have no conflicts of interests to declare.

1

## Supporting information

As a service to our authors and readers, this journal provides supporting information supplied by the authors. Such materials are peer reviewed and may be re‐organized for online delivery, but are not copy‐edited or typeset. Technical support issues arising from supporting information (other than missing files) should be addressed to the authors.

Supporting Information

## Data Availability

The data that support the findings of this study are available from the corresponding author upon reasonable request.
