# Supplementary material for: Au⋅⋅⋅H−X (X=N or C) Intramolecular Interactions in Gold (I)‐NHC Carbene Complexes with Potential Anticancer Properties: A Quantum Mechanical Study with Two Basis Sets
Source: ChemistryOpen. 2024 Jun 25;13(10):e202400140. doi: 10.1002/open.202400140 (PMC11457764; doi:10.1002/open.202400140)
Supplement: Supplementary file 1 — Supporting Information [file OPEN-13-e202400140-s001.pdf]

ChemistryOpen

Supporting Information

Au···H–X (X = N or C) Intramolecular Interactions in Gold (I)-NHC Carbene Complexes with Potential Anticancer Properties: A Quantum Mechanical Study with Two Basis Sets

Maria Benavides* and Elizabeth Granda

Supporting Information:

Figure S1: Laplacian relief maps of (a) $[Au(IPr)(Me_2ImS)]^+$, (b) $[Au(IPr)(EtImS)]^+$, and (c) $[Au(IPr)(ImS)]^+$.

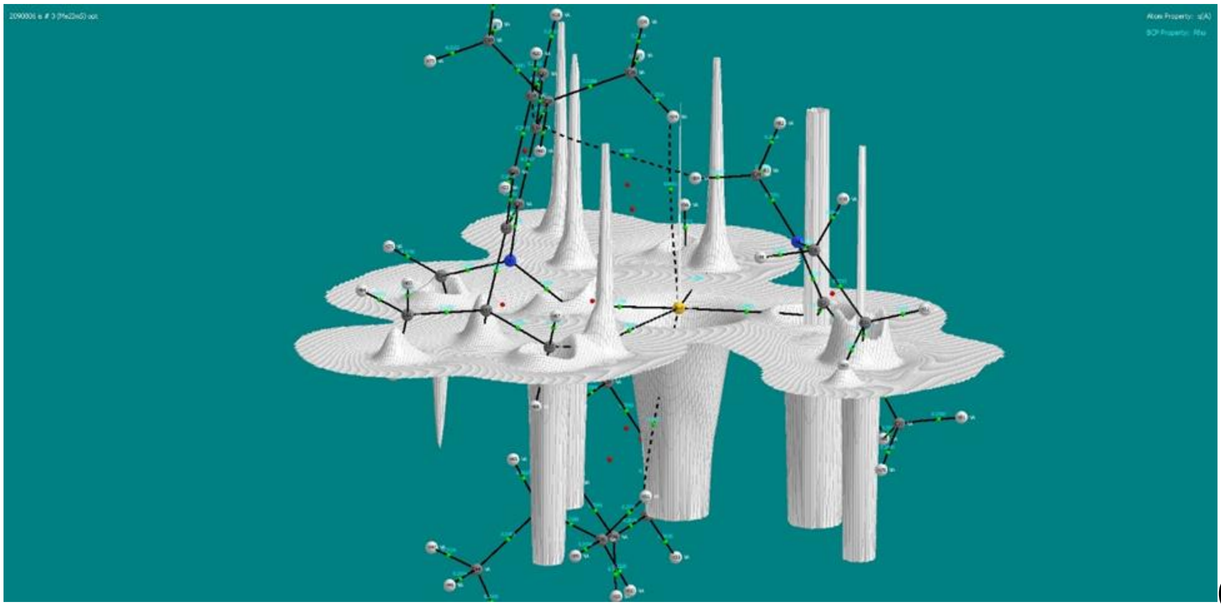

(a)

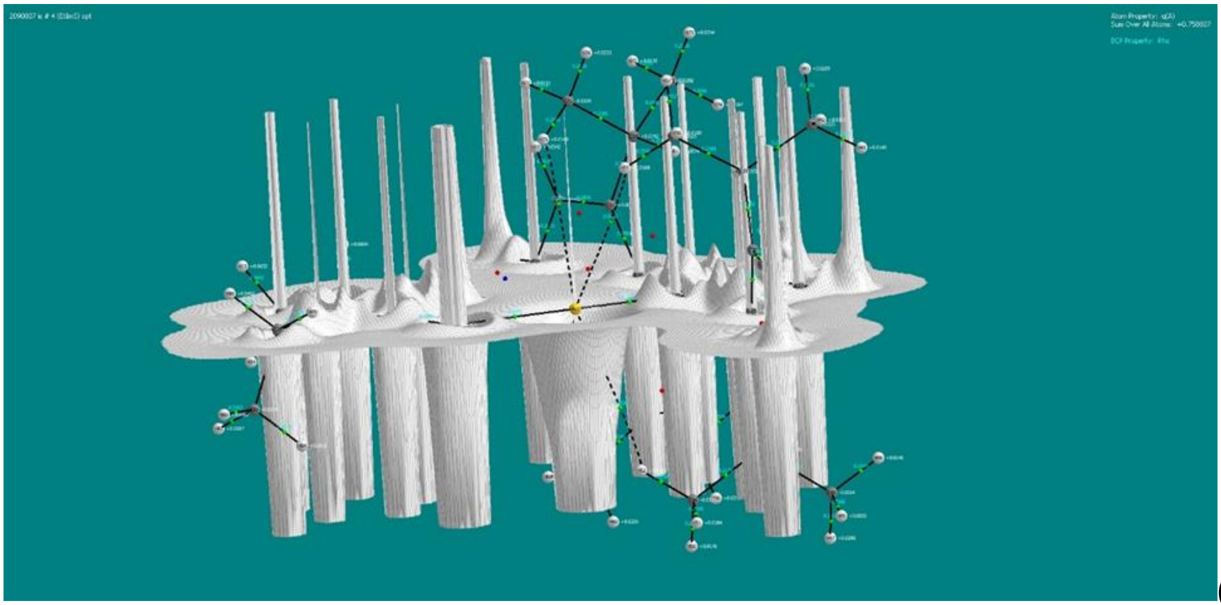

(b)

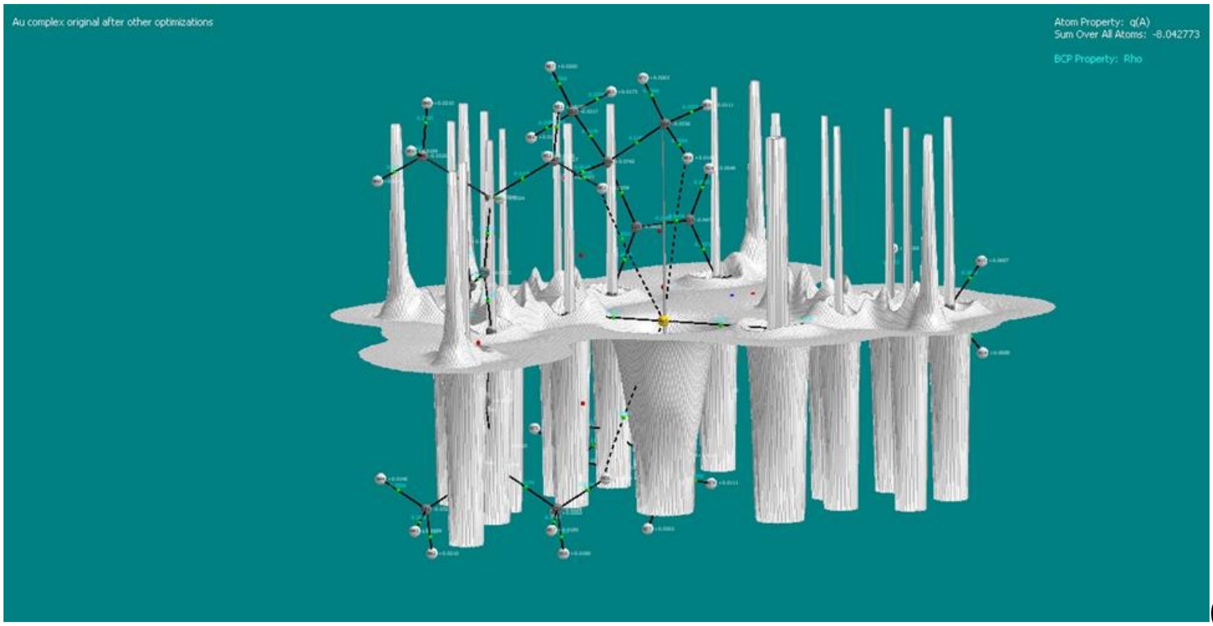

(c)

Figure S2: Molecular graphs with atomic charges and bond critical points of (a) $[Au(IPr)(Me_2ImS)]^+$, (b) $[Au(IPr)(EtImS)]^+$, and (c) $[Au(IPr)(ImS)]^+$.

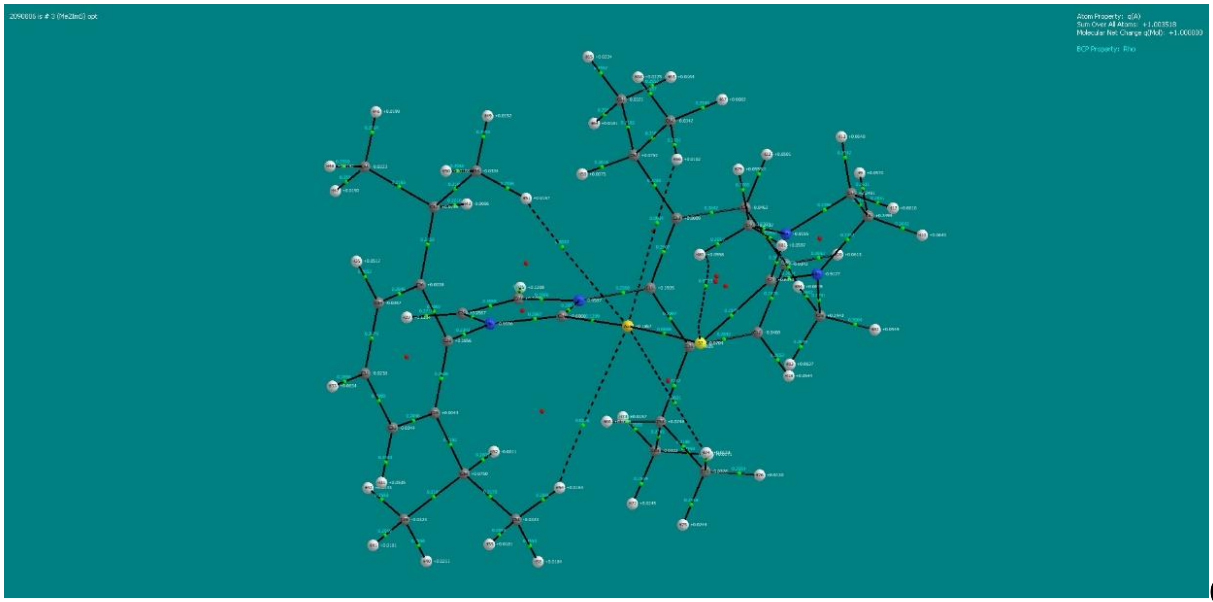

(a)

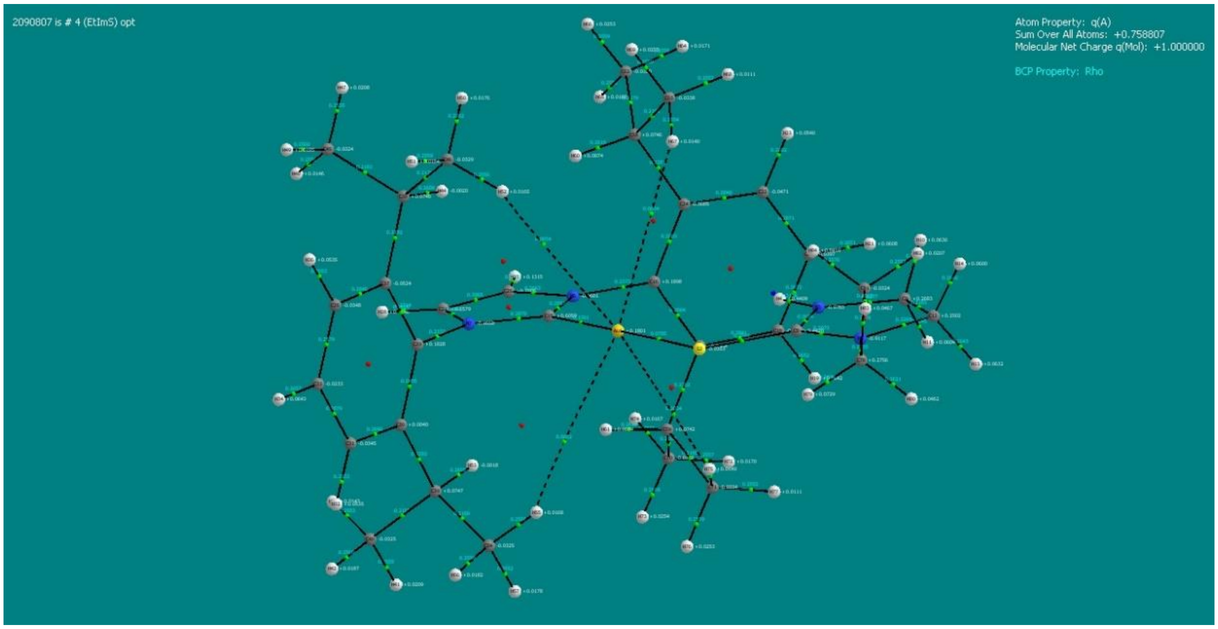
2090807 is # 4 (EtImS) opt
Atom Property: q(A)
Sum Over All Atoms: +0.758807
Molecular Net Charge q(Mol): +1.000000
BCP Property: Rho

(b)

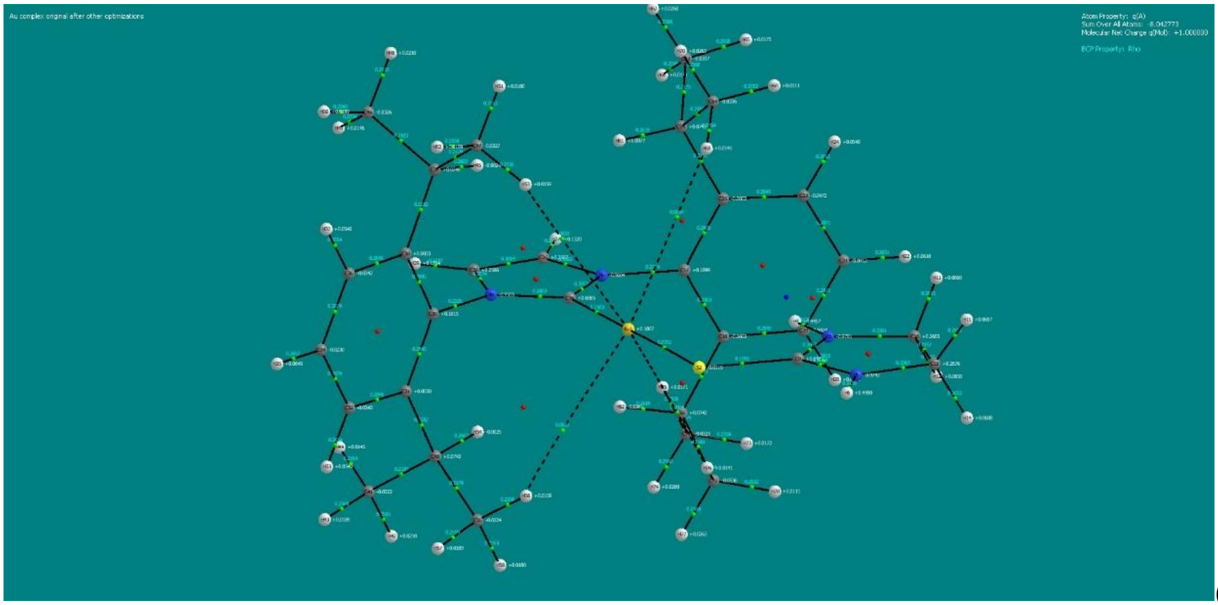
Au complex original after other optimizations
Atom Property: q(A)
Molecular Net Charge q(Mol): +1.000000
BCP Property: Rho

(c)

Figure S3: Computed IR spectra of (a) $[Au(IPr)(Me_2ImS)]^+$, (b) $[Au(IPr)(EtImS)]^+$, and (c) $[Au(IPr)(ImS)]^+$.

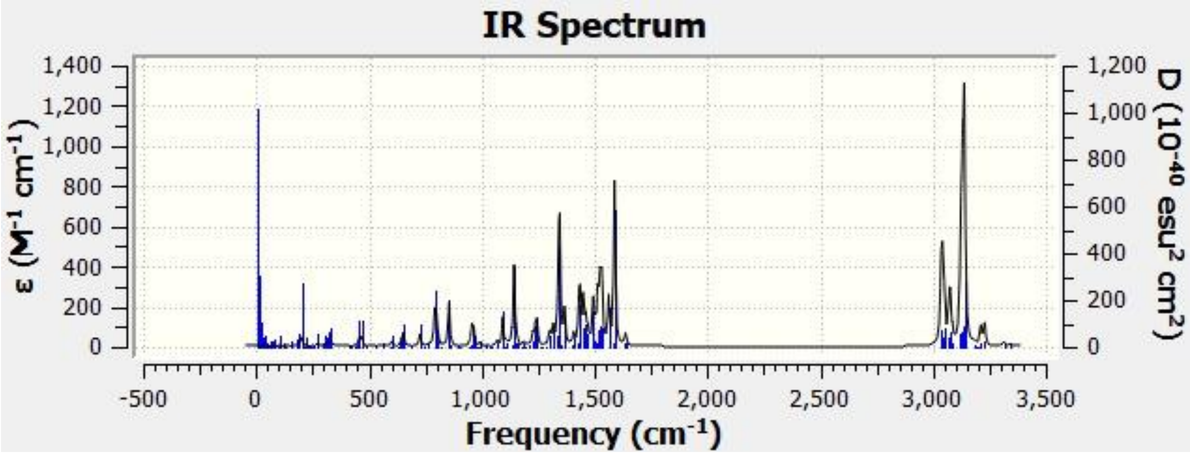

(a)

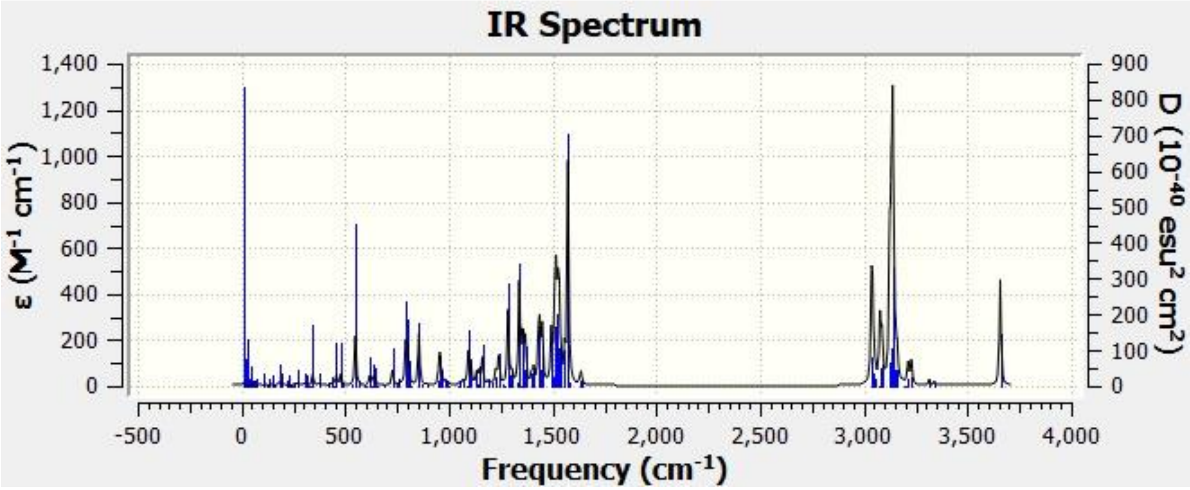

(b)

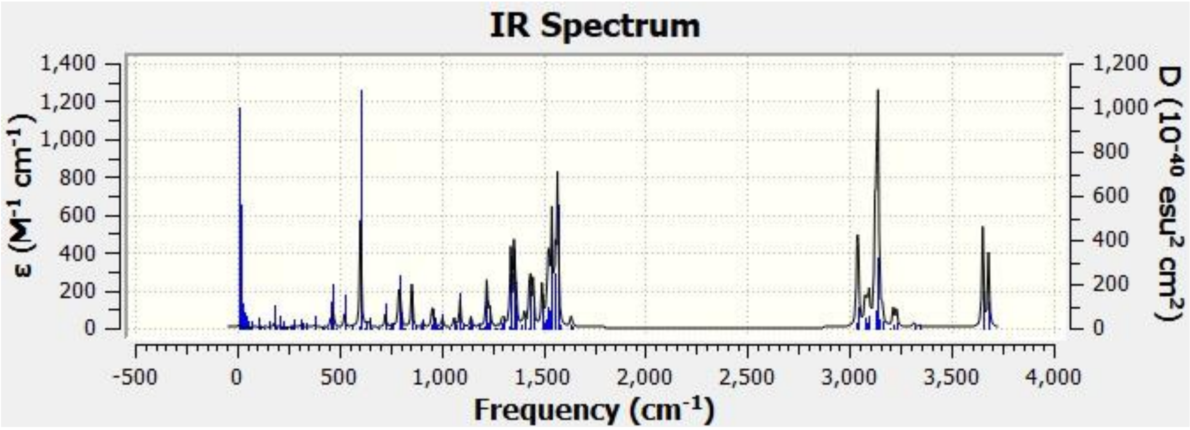

(c)
